# Insomnia, Inattention and Fatigue Symptoms of Women with Premenstrual Dysphoric Disorder

**DOI:** 10.3390/ijerph18126192

**Published:** 2021-06-08

**Authors:** Pai-Cheng Lin, Chih-Hung Ko, Yen-Ju Lin, Ju-Yu Yen

**Affiliations:** 1 Department of Psychiatry, Kaohsiung Municipal Ta-Tung Hospital, Kaohsiung Medical University, Kaohsiung 801, Taiwan; superfoxcat@yahoo.com.tw; 2 Department of Psychiatry, Kaohsiung Medical University Hospital, Kaohsiung Medical University, Kaohsiung 807, Taiwan; chihhungko@gmail.com (C.-H.K.); beyondfuture0610@gmail.com (Y.-J.L.); 3 Department of Psychiatry, Faculty of Medicine, College of Medicine, Kaohsiung Medical University, Kaohsiung 807, Taiwan; 4 Department of Psychiatry, Kaohsiung Municipal Siao-gang Hospital, Kaohsiung Medical University, Kaohsiung 812, Taiwan

**Keywords:** PMDD, insomnia, inattention, fatigue

## Abstract

Aim: Premenstrual dysphoric disorder (PMDD) has predictable, cyclic, psychological, and somatic symptoms, such as sleep problems. They result in functional impairment, are aggravated in the late luteal phase of the menstrual cycle, and are resolved by menstruation. The present study evaluated the insomnia, inattention, and fatigue symptoms of PMDD and their fluctuations during the menstrual cycle. Methods: A total of 100 women were diagnosed as having PMDD based on psychiatric interviews and a prospective investigation of three menstrual cycles. A total of 96 individuals without PMDD were recruited as controls. Their symptoms, namely insomnia, inattention, and fatigue as well as functional impairment were assessed by using the premenstrual symptoms screening tool, the Pittsburgh insomnia rating scale, the attention and performance self-assessment scale, and the fatigue-assessment scale during both premenstrual and follicular phases. Results: In both the premenstrual and follicular phases, women with PMDD experienced more severe insomnia, inattentiveness, and fatigue than did women in the control group. A paired *t*-test demonstrated that women with PMDD had more severe severity insomnia, inattentiveness, and fatigue in the luteal phase than in the follicular phase. A repeated-measures analysis of variance demonstrated that the interaction period of PMDD and a menstrual cycle was significantly associated with insomnia, inattentiveness, and fatigue. A further correlation analysis demonstrated that all three symptoms were positively associated with self-reported functional impairment due to PMDD. Conclusions: Our results demonstrated that women with PMDD experienced an exacerbation of insomnia, memory problems, difficulty maintaining focus, and fatigue in the premenstrual phase. These symptoms are correlated with PMDD symptoms severity and functional impairment, and as such, they should be evaluated, and interventions should be employed in the late luteal phase of women with PMDD.

## 1. Introduction

Premenstrual dysphoric disorder (PMDD) was included in the official diagnostic criteria of the Diagnostic and Statistical Manual of Mental Disorders, 5th Edition (DSM-5) in 2013 based on adequate support for its inclusion [1]. It is defined as predictable, cyclic, and functionally impairing psychological and somatic symptoms that are aggravated in the late luteal phase of the menstrual cycle and are resolved by menstruation [2]. The typical emotional presentations, such as irritability, depression, and anxiety, recur before menstruation and last for an average of six days per month during the majority of reproductive years. The cyclic pattern of physical and cognitive presentations, such as insomnia, inattention, and fatigue, have not been well evaluated in previous studies; whether these symptoms have the same mechanism or course of presentation is unclear.

Hypersomnia and are insomnia are the core symptoms of PMDD [3]. One study indicated that women with premenstrual syndrome (PMS) had poorer sleep quality, a higher perception of unrefreshing sleep, increased total sleep time recorded by polysomnography, a lower saturation of peripheral oxygen, and more subthreshold insomnia [4]. Another study that focused on female university students who experienced PMDD and sleep problems revealed that lower sleep quality, daytime dysfunction, and sleep disturbances were common among such students [5]. Sleep disturbance and decreased melatonin secretion due to hormonal fluctuations during the luteal phase of the menstrual cycle could explain the sleep complaints associated with PMDD [6]. 

In addition to sleep problems, fatigue and inattention deeply impair the function of women with PMDD. Lethargy and susceptibility to fatigue are also symptoms of PMDD. Studies have demonstrated that consistently higher progesterone levels during the luteal phase are associated with low levels of premenstrual fatigue [7]. Furthermore, women who experienced a sharp decline in progesterone during the luteal phase developed premenstrual symptoms, whereas women who experienced a gradual decline experienced no premenstrual dysphoria [8]. However, the fatigue symptoms across menstrual cycles among women with PMDD have not been adequately evaluated. 

Subjective difficulty in concentration is also a common complaint during the luteal phase for women with PMDD. Women with PMDD exhibited lower performance in immediate and delayed digit-recall tasks than women without PMDD [9]. The n-back task has also been used to evaluate the selective attention and working memory of women with PMDD. Women with PMDD had significantly poorer performance on two-back and three-back tasks in the luteal phase than the control group did [10]. Compared with women with mild or no PMS symptoms, women with moderate to severe PMS had significantly poorer accuracy and more errors of omission in the zero-back task as well as more errors of omission in the two-back task, indicating impairments in selective attention and working memory [11]. Cognitive deficits might contribute to inattention among women with PMDD. 

Because insomnia symptoms can be associated with fatigue and inattention [12,13], an evaluation of the associations between insomnia, fatigue, and inattention among women with PMDD might elucidate the mechanism of PMDD. Furthermore, how these symptoms affect the function of women with PMDD is not sufficiently understood. Thus, the aim of this study was to investigate the following: (1) the effects of menstrual cycle and PMDD on insomnia, inattention symptoms, and fatigue; (2) the associations between PMDD symptom severity, insomnia, inattention symptoms, and fatigue; and (3) the associations between these symptoms and functional impairment among women with PMDD.

## 2. Methods

### 2.1. Participants

Participants were recruited using an advertisement posted specifically for the untreated PMDD and control groups in a university campus from December 2013 to May 2015. Participants in the PMDD group were required to have positive responses to five or more of 11 DSM-5 criteria for PMDD [3], and most symptoms had to have dissipated after menstruation. The control group had positive responses to 11 DSM-5 criteria of PMDD or experienced no functional impairment with mild symptoms. To prevent the serotonin or estrogen function of participants from being affected by psychotropic or gonadotropic medications, participants taking such medications were screened and excluded from the study. A total of 240 women (142 in the PMDD group and 98 in the control group) with educational attainment of at least college level were interviewed by one of two psychiatrists to (1) exclude psychotic disorder and bipolar I disorder as possible causes for symptoms based on the results of the diagnostic interviews (Mini-International Neuropsychiatric Interview) and (2) diagnose PMDD based on DSM-5 criteria [3]. The PMDD group contained 137 participants, and the control group contained 96 participants (no PMDD). The informed consents were obtained from all participants. We prospectively followed all participants for three menstrual cycles. The study was approved by the Institutional Review Board of Kaohsiung Medical University Hospital (KMUHIRB-SV(I)-20150040).

### 2.2. Measures

#### 2.2.1. Premenstrual Symptoms Screening Tool 

The premenstrual symptoms screening tool (PSST) developed by Steiner and colleagues [14] is used to translate categorical DSM-IV criteria into a rating scale based on degrees of severity. The PSST contains 14 items rated on a 4-point scale that assess the severity of PMDD symptoms (1, not at all, to 4, severe) as well as five items rated on a 4-point scale that assess the degree of functional impairment in the premenstrual phase. We used the scale to screen women with moderate-to-severe premenstrual symptoms. They had to have (1) at least one of the first four of the 14 possible symptoms, (2) four or more of the 14 items that assess the severity of PMDD symptoms, and (3) at least one of the five items assessing functional impairments that were classified as moderate to severe [14]. The 14 items on symptom severity had a Cronbach’s alpha of 0.96, and the five items on functional impairment had a Cronbach’s alpha of 0.93. 

#### 2.2.2. PMDD Severity Questionnaire

The PSST is designed to assess symptoms only in the premenstrual phase; thus, we developed a PMDD severity questionnaire (PMDDSQ) to assess the severity of PMDD symptoms throughout the menstrual cycle and to determine which participants satisfied the criteria for the symptomatic cycle of PMDD during the study. The PMDDSQ is an 11-point questionnaire in which the severity of the 11 criteria for PMDD are rated, as described by the DSM-IV-TR. The severity of each symptom was rated from 0 (no symptoms at all) to 10 (extremely severe symptoms). The total PMDDSQ score represented PMDD symptom severity in the different phases of the menstrual cycle. The questionnaire had a Cronbach’s alpha of 0.98, and the 4-week test-retest reliability was 0.92 [15].

#### 2.2.3. Pittsburgh Insomnia Rating Scale, 20-Item Version

The 20-item version of the Pittsburgh insomnia rating scale (PIRS-20) is a self-report questionnaire derived from the original 65-item PIRS. It has 12 items on nighttime and daytime distress symptoms; 4 items on sleep parameters; and 4 items on quality, regularity, and depth of sleep to assess the patient’s sleep condition in the preceding week [16,17]. Each item is scored on a 4-point scale from 0 (not at all bothered) to 3 (severely bothered), with total scores ranging from 0 to 60, to provide an index of insomnia severity; a cut-off score of 20 was used to determine whether the respondent had clinical insomnia [17]. The PIRS-20 has a Cronbach’s alpha of 0.95 and a test-retest reliability of 0.92 [18]. 

#### 2.2.4. Attention and Performance Self-Assessment Scale

The 30-item attention and performance self-assessment (APSA) scale was initially developed to measure cognitive impairment in participants reporting subjective tinnitus [19]. Sequential principal factor analyses of the APSA scale resulted in a subscale that included 20 (APS20) of the original 30 items and two correlated subscales (AP-F1, AP-F2) with 9 items each. Each item had 5 response options and a recall period of 4 weeks. The response options were as follows: 0 (never), 1 (rarely), 2 (sometimes), 3 (often), and 4 (always). AP-F1 is predominantly used to measure prospective memory problems, whereas AP-F2 is used to measure difficulty maintaining focus [19]. The test-retest reliability of the APS20, AP-F1, and AP-F2 (intraclass correlation coefficients ≥ 0.87) subscales as well as the internal consistency (Cronbach’s alpha ≥ 0.89) are considered high [19]. We used the AP-F1 (prospective everyday memory problems) and AP-F2 (difficulties maintaining focus) subscales to evaluate inattentive symptoms, and higher scores indicated greater attention deficits.

#### 2.2.5. Fatigue-Severity Scale

The 9-item fatigue-severity scale (FSS) is, as indicated, used to measure the severity of fatigue caused by various conditions. This self-rating scale was developed by Krupp and colleagues [20]. Each item is rated from 1 to 7 depending on the extent to which the respondent agrees that an associated statement applied to them over the preceding week. A higher score represents greater fatigue in the previous week. The FSS has excellent internal consistency, demonstrated by a Cronbach alpha coefficient of 0.93, and it is a valuable tool for assessing and quantifying fatigue because it differentiates between healthy participants and patients with various diseases [21].

### 2.3. The Design of the Study and Its Procedures

In order to achieve the aims of the study, all participants took part in the investigation both in the premenstrual (within 1 week before menstruation, as predicted by the prior menstruation cycle) and follicular phases. The assessments included questionnaire evaluations for PMDD symptoms, functional impairment, insomnia, inattentive symptoms, and fatigue. Half of participants (69 in the PMDD group and 49 in the control group) were first assessed in the later-luteal phase and later assessed again in the follicular phase, and the remaining participants were assessed in the reverse manner. After the investigation was completed for the first menstrual cycle as described, we further arranged follow-ups for all participants with the PMDDSQ once a week for two menstrual cycles. According to the criteria for symptomatic cycles of PMDD [22], women with PMDD should have scores in the premenstrual phase that are 30% higher than their individual minimal menstrual-cycle scores. Participants were required to fulfill these criteria for two consecutive menstrual cycles to be entered in the PMDD group.

### 2.4. The Data Analysis

The independent *t*-test evaluated the difference in age and the scores of PSST, PIRS-20, APSA, and FSS between PMDD group and controls. The paired *t*-test evaluated the difference in the scores of PIRS-20, APSA, and FSS between late luteal phase and follicular phase among PMDD group and among control group. Repeated-measures analysis of variance (ANOVA) was used to evaluate insomnia, inattentive symptoms, and fatigue as functions of the menstrual cycle phase (late luteal versus follicular) and PMDD diagnosis to demonstrate their exacerbation in the PMDD group. Then, the Pearson’s correlation coefficient evaluated the association between PMDD symptoms severity, insomnia, inattentive symptoms, and fatigue in late luteal phase among women with PMDD. Lastly, the forward linear regression model was used to regress functional impairment of women with PMDD against their insomnia, inattentive symptoms, and fatigue with control in age and educational level. 

## 3. Results

A total of 37 asymptomatic women were excluded from the PMDD group because they did not meet the criteria of having two consecutive symptomatic cycles [22] according to the prospective investigation completed using the PMDDSQ. A total of 100 women with PMDD and 96 control participants fulfilled the recruitment criteria. No significant differences in age or educational level between the PMDD and control groups were observed (Table 1). 

### 3.1. Premenstrual Changes in Insomnia, Inattentive Symptoms, and Fatigue

The results demonstrate that women with PMDD had a higher incidence of insomnia, prospective everyday memory problems, difficulty maintaining focus, and fatigue in both premenstrual and follicular phases (Table 1). A paired *t*-test demonstrated that women with PMDD experienced relatively more insomnia (t = 4.12, *p* < 0.001), prospective everyday memory problems (t = 3.64, *p* < 0.001), difficulty maintaining focus (t = 3.80, *p* < 0.001), and fatigue in the premenstrual phase (t = 5.83, *p* < 0.001). A repeated-measures two-factor analysis of variance (ANOVA) (Table 2) revealed the exacerbation of insomnia (F = 6.12, *p* = 0.01), prospective everyday memory problems (F = 8.51, *p* = 0.004), difficulty maintaining focus (F = 9.88, *p* = 0.002), and fatigue (F = 15.76, *p* < 0.001) in women with PMDD than in control group participants. 

### 3.2. Associations Between PMDD Symptom Severity, Insomnia, Inattentive Symptoms, and Fatigue 

Pearson’s correlation coefficient (Table 3) revealed a significant association between PMDD symptom severity, insomnia, prospective everyday memory problems, difficulty maintaining focus, and fatigue among women with PMDD in the luteal phase. 

### 3.3. Association Between Insomnia, Inattentive Symptoms, Fatigue, and Functional Impairment among Women with PMDD

The result demonstrated a significant association between insomnia, inattentive symptoms, fatigue, and functional impairment in women with PMDD. Furthermore, a linear regression analysis (Table 4) indicated that fatigue was the first factor in the regression model (B = 0.23, t = 2.25, *p* = 0.03), followed by prospective everyday memory problems (B = 0.21, t = 2.10, *p* = 0.04). The regression model significantly predicted the functional impairment (F = 4.98; *p* = 0.001). The variance inflation fact value (1.14–1.22) and Durbin–Watson value (2.16) did not indicate multi-collinearity and auto-correlation in the residuals, respectively.

## 4. Discussion

The results of this study revealed that women with PMDD had poor sleep quality, inattention, and fatigue in the premenstrual phase and follicular phase. However, the results also indicated that symptoms can be mild in the follicular phase, with little functional impairment, but worsen significantly in the late luteal phase. 

### 4.1. PMDD-Associated Fatigue

Our study demonstrated that fatigue had a strong effect on the functional impairment of women with PMDD, especially in the premenstrual phase, and this suggests that the fluctuation of ovarian hormones plays a role in the fatigue in PMDD. In aforementioned studies, consistently higher progesterone levels during the luteal phase were associated with low levels of premenstrual fatigue symptoms, and a sharp decline in progesterone level in the luteal phase was associated with the development of premenstrual symptoms [7,8]. Moreover, studies have demonstrated that decreases in estrogen cause the hypothalamus to release norepinephrine and trigger a decline in serotonin, acetylcholine, and dopamine, in turn leading to insomnia, depression, and fatigue—the typical symptoms of PMDD [23]. Another study also reported that mice with ovariectomies could restore physical activity, and prevent muscles fatigue through the combined treatment of estradiol and progesterone (though predominantly driven by estradiol) [24]. These results suggested that PMDD fatigue is associated with a decrease in estrogen and progesterone, especially in the premenstrual phase; further studies are required to investigate the relationships between estrogen, progesterone, and fatigue in women with PMDD during different phases of the menstrual cycle.

### 4.2. Inattentive Symptoms Associated with PMDD

Attention deficit often occurs in women with PMDD. Reed and her colleague found that women with PMDD had impaired performance in immediate and delayed word recall tasks, immediate and delayed digit recall tasks, and the digit-symbol substitution test compared with women in the control group during the luteal phase [9]. Moreover, Slyepchenko and her colleague observed impaired performance on the n-back test among women with moderate-to-severe PMS compared with women with mild PMS symptomatology during the asymptomatic follicular phase of the menstrual cycle; this indicated impairment of selective attention and working memory [11]. Despite these findings, no study has compared the attention problems associated with the different phases of the menstrual cycle or discussed the association between attention and functional impairment among women with PMDD. In our study, women with PMDD were observed to have greater difficulty maintaining attention during the premenstrual phase than during the follicular phase, and because their functional impairment was related to inattention, prospective everyday memory problems resulted. This may be related to changes in estradiol (E2) levels during the menstrual cycle. Functional MRI studies with numerous paradigms suggested that brain-function efficiency improved during a sustained attention task among postmenopausal women who received E2, and during a working-memory task, postmenopausal women had increased frontal lobe activity as task difficulty increased following treatment with E2 [25]. Further studies are required to investigate the association between estradiol and attention during the different phases of the menstrual cycle in women with PMDD. 

### 4.3. Sleep Problems Associated with PMDD

Studies have demonstrated that women with PMDD have more sleep problems than those who do not, including bed time, sleep quality, sleep-onset latency, sleep maintenance, and wake time [6]. However, no study has investigated the sleep conditions of women with PMDD during different phases of the menstrual cycle. Our study demonstrated that women, especially those with PMDD, have poorer sleep during the premenstrual phase than during the follicular phase. Moreover, the severity of insomnia was greater in the PMDD group than in the control group during both phases. These results may be related to the role of hormones in sleep outcomes. In their luteal phase, women with PMDD exhibited a decreased response to melatonin compared with in their follicular phase, which may have deregulated circadian rhythm changes, increased progesterone levels, and decreased levels of allopregnenalone, thus increasing levels of gamma aminobutyric acid, which are responsible for both mood and sleep disturbance [6]. Moreover, studies have indicated that a decrease in estrogen triggers a decline of serotonin, acetylcholine, and dopamine, thus leading to insomnia [23]. Further studies on the mechanisms behind these phenomena are required to clarify the relationships between melatonin, progesterone, estrogen, serotonin, and sleep problems among women with PMDD.

### 4.4. Clinical Implications

A significant association exists between insomnia, attention difficulty, fatigue, and functional impairment among women with PMDD. Furthermore, fatigue is the factor most associated with functional impairment among women with PMDD, whereas prospective everyday memory problems are secondary to it, according to the results of a linear regression analysis. Therefore, determining how to overcome fatigue and inattention symptoms, such as prospective everyday memory problems, may be key to maintaining function among women with PMDD during the late luteal phase.

Several approaches can be taken to reduce the symptoms of PMDD. Lifestyle changes, such as increasing physical activity, have been demonstrated to alleviate symptoms, such as depressed mood, fatigue, bloating, and constipation, which are also frequently observed in women with PMDD; however, strong evidence to support the recommendation is scant [26]. Dietary changes, such as reducing alcohol, caffeine, and sugar intake and increasing complex carbohydrate intake during the luteal phase, may alleviate PMDD symptoms; these changes are frequently recommended, but systematic evaluations of their efficacy are lacking [26]. Vitamin D supplementation benefited women with PMDD who experienced premenstrual symptoms of cramps, fatigue, anxiety, desire to be alone, and confusion; a clinical trial showed that these symptoms are related to inadequate vitamin D intake [27]. Nutritional supplementation with calcium, vitamin B6 [28], and omega 3 fatty acids [29] have been effective in improving premenstrual symptoms. Psychological interventions, such as cognitive behavioral therapy (CBT) and Internet-based CBT, are also effective for reducing symptoms of PMDD [30].

A repeated-measures ANOVA suggested that women with PMDD had significantly increased severity of insomnia, inattention, and fatigue during the late luteal phase, and this phenomenon suggests that these symptoms should be evaluated and interventions should be employed during the late luteal phase. Moreover, only a few studies have examined the functional performance changes among women with PMDD. Baller and colleagues observed that abnormal working memory activation in people with PMDD, specifically in the dorsolateral prefrontal cortex, was related to PMDD severity, symptoms, age at onset, and condition burden [31]. In another study, cognitive control and working memory declined during the premenstrual phase among women with PMDD, and the G/G genotype of HTR1A (rs6295) was reported to be associated with impaired working memory during the premenstrual phase and the premenstrual decline of cognitive function [10]. With the exception of how inattention and working-memory deficits affect the functional impairment of women with PMDD, no studies have discussed the role that insomnia and fatigue play in the impaired performance of women with PMDD. Our study demonstrated that not only inattention and working memory but also insomnia and fatigue were correlated with functional impairment among women with PMDD during the late luteal phase. Therefore, these symptoms should be evaluated, and related interventions, such as the cognitive-behavioral therapy for insomnia, exercise, or pharmacotherapy [32], should be employed during the late luteal phase.

The present study had several notable limitations. First, although many candidates were excluded based on the criteria for symptomatic menstrual cycles [22] during the prospective investigation, the more numerous PMDD group could have notably increased the power of the results. Second, the cross-sectional nature of this study meant that we could not confirm the causal relationships between fatigue, sleep problems, attention, and PMDD.

## 5. Conclusions

Our results demonstrated that women with PMDD experienced more severe insomnia, prospective everyday memory problems, difficulty maintaining focus, and fatigue than healthy controls, and their symptoms exacerbated in the premenstrual phase. These symptoms are associated with both PMDD symptoms severity and functional impairment among women with PMDD, and, therefore, they should be evaluated and treated during the late luteal phase of the menstrual cycle.

## Figures and Tables

**Table 1:** The insomnia, inattentive symptoms, and fatigue in late luteal and follicular phase among women with PMDD and controls.

Variables (Missing Number)	PMDD Group (mean ± SD) (N = 100)	Paired *t*-Test	Control Group (mean ± SD) (N = 96)	Paired *t*-Test	Independent *t*-Test
**Age**	24.77 ± 3.32		24.84 ± 3.46		−0.152
**PMDD symptoms**	33.96 ± 6.10		19.24 ± 4.36		19.499 ***
**Functional impairments**	10.95 ± 2.68		5.98 ± 1.27		16.674 ***
**Pittsburgh insomnia rating scale (PIRS)**					
Late luteal	25.19 ± 10.16	4.124 ***	10.81±7.19	2.153 *	11.476 ***
Follicular	20.96 ± 11.06		9.55 ± 6.25		8.901 ***
**Prospective everyday memory problems**					
Late luteal	17.14 ± 5.64	3.636 ***	10.83 ± 4.75	0.260	8.427 ***
Follicular	14.91 ± 5.82		10.72 ± 4.95		5.406 ***
**Difficulties keeping attention focus**					
Late luteal	17.72 ± 5.59	3.802 ***	10.11 ± 4.88	0.265	10.077 ***
Follicular	15.20 ± 6.42		9.98 ± 5.13		6.294 ***
**Fatigue-severity scale**					
Late luteal	44.53 ± 8.42	5.827 ***	28.04 ± 9.71	0.670	12.641 ***
Follicular	38.77 ± 11.14		27.44 ± 10.13		7.418 ***

*: *p* < 0.05; ***: *p* < 0.001.

**Table 2:** The repeated measures ANOVA for insomnia, inattentive symptoms, and fatigue as functions of the menstrual-cycle phase (late luteal versus follicular) and PMDD diagnosis (N = 196).

		With-Subject Analysis	
	df	Mean Square	F
**Pittsburgh insomnia rating scale (PIRS)**			
MC (luteal versus follicular)	1	767.39	21.28 ***
PMDD (PMDD versus controls)	1	16,254.07	130.64 ***
MC * PMDD	1	220.59	6.12 *
**Prospective everyday problems**			
MC (luteal versus follicular)	1	152.199	10.96 **
PMDD (PMDD versus controls)	1	2650.44	61.99 ***
MC * PMDD	1	118.251	8.51 **
**Difficulties keeping attention focus**			
MC (luteal versus follicular)	1	190.980	11.95 **
PMDD (PMDD versus controls)	1	4002.37	88.37 ***
MC * PMDD	1	157.902	9.88 **
**Fatigue-severity scale**			
MC (luteal versus follicular)	1	1006.978	23.13 ***
PMDD (PMDD versus controls)	1	18,547.39	121.07 ***
MC * PMDD	1	686.232	15.76 ***

*: *p* < 0.05; **: *p* < 0.01; ***: *p* < 0.001.

**Table 3:** The correlation between PMDD symptoms severity, insomnia, inattentive symptoms, and fatigue among women with PMDD (N = 100).

	PIRS	PEMP	DKAF	FSS
PMDD severity	0.349 **	0.431 **	0.380 **	0.312 **
Functional impaired	0.250 *	0.313 **	0.257 **	0.306 **
PIRS	1	0.222 *	0.245 *	0.357 **
PEMP		1	0.705 **	0.398 **
DKAF			1	0.426 **

*: *p* < 0.05; **: *p* < 0.01. PIRS: Pittsburgh insomnia rating scale. PEMP: Prospective everyday memory problems. DKAF: Difficulties keeping attention focus. FSS: Fatigue severity scale.

**Table 4:** The forward linear regression model for the association between insomnia, inattentive symptoms, fatigue, and functional impairment among women with PMDD (N = 100).

Variables	B	SE	*β*	t	Tolerance	VIF
Functional Impairments						
Age	0.15	0.08	0.19	1.89	0.87	1.14
Educational level	−0.29	0.22	−0.12	−1.35	0.88	1.14
FSS	0.07	0.03	0.23	2.25 *	0.82	1.22
PEMP	0.10	0.05	0.21	2.10 *	0.84	1.20
F = 4.98 **; R^2^ = 16.7%; Durbin–Watson value = 2.16						

*: *p* < 0.05; **: *p* < 0.01; FSS: Fatigue-severity scale. PEMP: Prospective everyday memory problems. VIF: Variance inflation fact.

## Data Availability

The data presented in this study are available on request from the corresponding author. The data are not publicly available due to privacy.
